# Screening and identification of *Theileria annulata* subtelomere-encoded variable secreted protein-950454 (SVSP454) interacting proteins from bovine B cells

**DOI:** 10.1186/s13071-021-04820-4

**Published:** 2021-06-11

**Authors:** Zhi Li, Junlong Liu, Quanying Ma, Aihong Liu, Youquan Li, Guiquan Guan, Jianxun Luo, Hong Yin

**Affiliations:** 1 State Key Laboratory of Veterinary Etiological Biology, Key Laboratory of Veterinary Parasitology of Gansu Province, Lanzhou Veterinary Research Institute, Chinese Academy of Agricultural Science, Xujiaping 1, Lanzhou, Gansu, 730046 People’s Republic of China; 2 Jiangsu Co-Innovation Center for Prevention and Control of Important Animal Infectious Diseases and Zoonoses, Yangzhou, 225009 People’s Republic of China

**Keywords:** *Theileria annulata*, SVSP454, Yeast two-hybrid, Co-IP, BiFC, CCDC181, MRPL30

## Abstract

**Background:**

*Theileria annulata* is a protozoan parasite that can infect and transform bovine B cells, macrophages, and dendritic cells. The mechanism of the transformation is still not well understood, and some parasite molecules have been identified, which contribute to cell proliferation by regulating host signaling pathways. Subtelomeric variable secreted proteins (SVSPs) of *Theileria* might affect the host cell phenotype, but its function is still not clear. Therefore, in the present study, we explored the interactions of SVSP454 with host cell proteins to investigate the molecular mechanism of *T. annulata* interaction with host cells.

**Methods:**

The transcription level of an SVSP protein from *T. annulata*, SVSP454, was analyzed between different life stages and transformed cell passages using qRT-PCR. Then, SVSP454 was used as a bait to screen its interacting proteins from the bovine B cell cDNA library using a yeast two-hybrid (Y2H) system. The potential interacting proteins of host cells with SVSP454 were further identified by using a coimmunoprecipitation (Co-IP) and bimolecular fluorescence complementation (BiFC) assays.

**Results:**

SVSP454 was transcribed in all three life stages of *T. annulata* but had the highest transcription during the schizont stage. However, the transcription level of SVSP454 continuously decreased as the cultures passaged. Two proteins, *Bos Taurus* coiled-coil domain 181 (CCDC181) and *Bos Taurus* mitochondrial ribosomal protein L30 (MRPL30), were screened. The proteins CCDC181 and MRPL30 of the host were further identified to directly interact with SVSP454.

**Conclusion:**

In the present study, SVSP454 was used as a bait plasmid, and its prey proteins CCDC181 and MRPL30 were screened out by using a Y2H system. Then, we demonstrated that SVSP454 directly interacted with both CCDC181 and MRPL30 by Co-IP and BiFC assays. Therefore, we speculate that SVSP454-CCDC181/SVSP454MRPL30 is an interacting axis that regulates the microtubule network and translation process of the host by some vital signaling molecules. Identification of the interaction of SVSP454 with CCDC181 and MRPL30 will help illustrate the transformation mechanisms induced by *T. annulata*.

**Graphic abstract:**

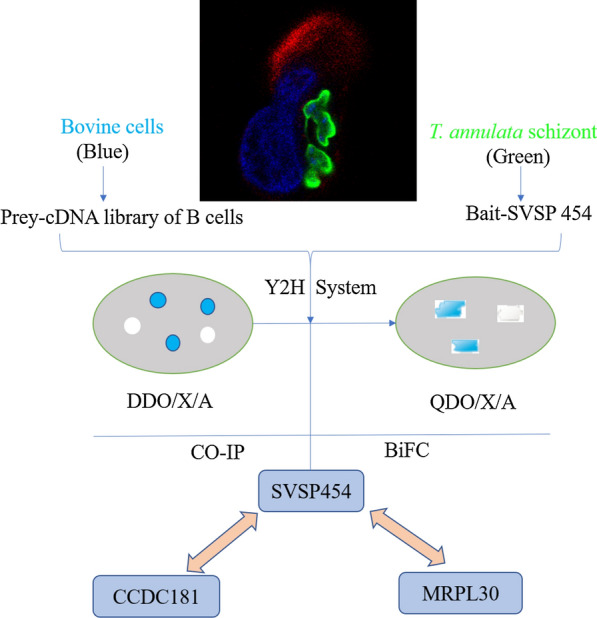

## Background

*Theileria annulata* is an intracellular apicomplexan parasite transmitted by *Hyalomma* tick species, and is the pathogen responsible for tropical theileriosis. Tropical theileriosis causes more than 1.1 million cattle to die and approximately hundreds of millions of dollars of losses per year in tropical and subtropical areas [1]. *Theileria annulata* together with *T. parva* are referred to as “transforming species” for their ability to induce the uncontrolled proliferation of infected cells [2]. The target cells of these two *T.* species are different, where *T. annulata* mainly infects B cells, macrophages and dendritic cells, while *T. parva* invades T and B lymphocytes. However, infection of both parasites could cause acute lymphoproliferative disease, which shares some clinical characteristics with human leukemias [3]. In addition to uncontrolled proliferation, the cells transformed by *T. annulata* also have other cancer hallmarks, such as increased metastasis and invasiveness and deregulated cellular energetics [3–6]. The transformation induced by *T. annulata* is reversible, once the cells are treated with a theilericidal drug, they lose cancer-like characteristics and undergo the programmed apoptosis [7].

To drive host cell transformation, some molecules of *T. annulata* have been confirmed to highjack host signaling pathways that contribute to the immortality of cells, such as EB1 and TaPin1 [8–10]. However, the detailed mechanisms by which *T. annulata* manipulates the host cell and by which parasitic molecules are responsible for cancer-like phenotypes remain largely unknown. To understand the mechanisms of the transformation induced by *Theileria*, the genome of *T. annulata* was sequenced, and 3792 putative proteins were predicted [11]. The proteins expressed at the macroschizont stage and released into the cell cytoplasm or expressed on the surface of parasites are possibly involved in the process of transformation [11]. *Theileria annulata* schizont AT-hook protein (TashAT) family proteins, which matched these criteria, were confirmed to localize in the host cell nucleus and affected the host cell phenotype and proliferation [3, 12]

The SVSP multigene family, another large gene family, is mainly expressed at the schizont stage of both *T. parva* and *T. annulata*, and has been suspected to be involved in host cell transformation, invasion, and immune evasion [3, 13, 14]. Most SVSP genes are distributed across the subtelomeres, and have unique features, including diversity in length, atypical codon usage and extensive nucleotide variability. Moreover, the majority of SVSPs have signal peptides and are localized in the nuclear and cytoplasmic regions of host cells [11, 15], which makes it more likely that SVSP molecules interfere with host cell signaling “hubs” to regulate host cell functions.

Therefore, in the present study, we explored the interactions of SVSP proteins and host cell proteins. First, the mRNA level of SVSP454 was compared between *T. annulata* at different life stages and transformed cells at different passages with qPCR. Then, we used SVSP454 as the bait plasmid to screen the interacting proteins of the host by a yeast two-hybrid system. We demonstrated that SVSP454 interacts with both CCDC181 and MRPL30 using a coimmunoprecipitation (Co-IP) assay. With a bimolecular fluorescence complementation (BiFC) assay, the interaction of SVSP454 with CCDC181 and MRPL30 was confirmed in the cell context. The subcellular colocalization of the interacting proteins indicated that their interaction occurs in the intracellular compartments. In addition, flow cytometry was used to observe the median fluorescence intensity (MFI) emitted during the interactions between bait and prey proteins.

## Methods

### Cell culture

*T. annulata* schizont-infected cell lines were provided by the Vector and Vector-borne Disease (VVBD) laboratory, Lanzhou Veterinary Research Institute (LVRI), China. Cells were cultured in RPMI 1640 medium (Biological Industries, Kibbutz Beit Haemek, Israel) plus 10% fetal bovine serum (Biological Industries, Kibbutz Beit Haemek, Israel) and 100 mg/ml penicillin/streptomycin, and maintained at 37 °C in an incubator with 5% CO_2_. HEK293T cells were obtained from the China Center for Type Culture Collection, Shanghai, China, and cultured in Dulbecco's Modified Eagle Medium (DMEM) (Gibco, New York, USA) containing 10% fetal bovine serum (Gibco, New York, USA).

### Quantitative real-time polymerase chain reaction (qRT-PCR)

Total RNA of *T. annulata-*infected cells at different passages (F10, F20, F55, F110, and F165), and the RNA for suspensions of sporozoite and merozoite for *T. annulata* infection were extracted using a RNeasy Mini kit (QIAGEN, Dusseldorf, Germany) according to the user manual. cDNA was then synthesized via a PrimeScript™ RT Reagent Kit with gDNA Eraser (Perfect Real Time) (Takara, Dalian, China). The specific primers of SVSP454 (Accession No: XM950454.1) were as follows: SVSP454-F (5′-TAAAGGGAAATGGGTGTCTA-3′) and SVSP454-R (5′-CACTTGTGCTAAGGCTAACG-3′). qPCR reactions were carried out in a final volume of 20 μL containing 10 μL TB Green® Premix Ex Taq™ II (Tli RNaseH Plus) (2 ×) (TaKaRa, Dalian, China), 10 μM of each primer, 0.4 μL ROX Reference Dye II (50 ×) (TaKaRa, Dalian, China), 2 μL of cDNA template and 10 μL Easy Dilution for Real Time PCR System (TaKaRa, Dalian, China). And the cDNA from PBMC for bovine uninfected *T. annulata* was used as the negative control in the qPCR system. The qPCR protocol composed of initial denaturation at 95 °C for 30 s followed by 40 cycles of 2 step PCR (95 °C for 5 s, 60 °C for 34 s) and dissociation stage (95 °C for 15 s, 60 °C for 1 min, 95 °C for 15 s). And qRT-PCR was amplified with Agilent Technologies Stratagene MX3005P Thermocycler (California, USA). *T. annulata* transcription levels of β-actin were used for normalization. The specific primers for β-actin of *T. annulata* were β-actin-F (5′-GAGACCACCTACAACAGCATCATG-3′) and β-actin-R (5′-CACCTTGATCTTCATGGTGCTGGG-3′). The relative transcription levels of SVSP454 were determined using the comparative cycle threshold (2^−ΔΔ*CT*^) Ct method.

### Western blotting

The cells were lysed using RIPA lysis buffer (Beyotime, #P0013B) containing a phosphatase inhibitor cocktail (Roche, #4906845001) and protease inhibitor (Roche, #4693132001) and incubated on ice for 20 min. Following the lysis step, the cell lysates were centrifuged at 14,000×*g* for 10 min at 4 °C to collect the supernatants. The protein concentration was determined by using the Pierce™ BCA Protein Assay Kit (Thermo Fisher Scientific, #23225). Equal amounts of proteins were separated by 12% SDS-PAGE and transferred onto a PVDF membrane (Millipore, Massachusetts, USA). The PVDF membrane was blocked with 1 × TBST buffer containing 5% BSA (Amresco, Washington, USA) for 2 h at room temperature (RT). After washing three times with TBST buffer for 5 min each time, primary antibodies that were mouse anti-FLAG tag monoclonal antibody (Sigma, #F1804) or rabbit anti-MYC tag monoclonal antibody (CST, #2278S) were added and incubated overnight at 4 °C. Following washing, the membrane was probed with HRP-conjugated goat anti-mouse (Abcam, #ab6789) or donkey anti-rabbit IgG (Abcam, #ab6701) antibodies by gentle shaking at RT for 1 h. Finally, the membrane was washed with TBST four times, and the proteins on the membrane were detected using SuperSignal™ West Pico PLUS Chemiluminescent Substrate (Thermo Fisher Scientific, #34577). A GAPDH loading control monoclonal antibody (GA1R) derived from mice was purchased from Thermo Fisher Scientific (#MA5-15738), and served as a standard to evaluate the protein levels.

### Subcellular localization of the SVSP454 protein in *T. annulata*-infected cells

*T. annulata-*infected cells (2.0–3.0 × 10^5^) at different passages (F10, F20, F55, F110, and F165) were seeded on glass slides (NEST) in a 12-well cell culture plate. After culturing for 24 h, the cells were washed twice with PBS and then fixed with 4% paraformaldehyde at room temperature for 30 min. After three washes with PBS, the cells were permeabilized with 0.5% Triton X-100 for 10 min at room temperature. Following three washes, the cells were blocked with 3% bovine serum albumin (BSA) in PBS at 37 °C for 1 h. For the immunofluorescence assay, after washing the plate three times, the cells were incubated with 500 μL primary antibody against SVSP454 derived from rabbit (data not shown) at a 1:100 dilution in PBS with 1% BSA at 37 °C for 1 h. After five washes with PBS, the cells were stained with an Alexa Fluor 488 secondary antibody (Life Technologies, USA) at a 1:1000 dilution for 1 h at 37 °C. Following four washes, the cells were incubated with Hoechst 33342 (Life Technologies, USA) at a 1:2000 dilution for nuclear staining at 37 °C for 10 min. After washing out the remaining fluorochrome with PBS, the cells were then incubated with Alexa Fluor™ 594 Phalloidin for cytoskeleton staining (Life Technologies, USA) at a 1:100 dilution for 30 min at 37 °C. After washing, the cells were observed under a confocal microscope (Leica TCS) with a 63× oil objective. At least 100 random cells per slide were checked, and the most representative images from each slide were selected for presentation. Moreover, the subcellular location of SVSP454 in *T. annulata*-infected cell lines was predicted using CELLO v.2.5, a subCELlular LOcation predictor (http://cello.life.nctu.edu.tw/).

### Construction and analysis of the bait plasmid

The fragment of the target gene was amplified with PCR from the cDNA of *T. annulata*-infected cells. The specific primers were designed based on the reference sequence of SVSP950454 (Accession No: XM950454, SVSP454), and the nucleotide sequences were: SVSP454-F (5′-CCGGAATTCATGAATAAATATATAACATAT-3′) and SVSP454-R (5′-TGCACTGCAGATCCTCCGATTTATCATATTT-3′) with the restriction site underlined. The final volume of PCR reaction was 50 μL, which contained 10 μL 5× PrimeSTAR GXL Buffer (TaKaRa, Dalian, China), 4 μL dNTP Mixture (2.5 mM), 20 μM of each primer, 1 μL PrimeSTAR GXL DNA Polymerase (TaKaRa, Dalian, China), 4 μL of cDNA template, and 17 μL ddH_2_O. The protocol of PCR reactions was performed as follows: 35 cycles of denaturation (98 °C for 10 s, 50 °C for 15 s, 68 °C for 20 s) and the final extension at 68 °C for 10 min. The PCR products were purified by using a Cycle-Pure Kit (OMEGA Bio-Tek, USA), which was followed by digestion with restriction enzymes *EcoRI* and *PstI* (Thermo Fisher Scientific, #FD0274 and #FD0614). The products were then ligated to the pGBKT7 plasmid, which was digested with the same enzymes as previously described. Finally, the recombinant SVSP454-pGBKT7 plasmid was further identified using double restriction enzyme digestion and sequencing (Sangon Biotech, Shanghai, China), and only the positive plasmid was used as the bait plasmid for further experiments.

### Autoactivation and toxicity determination of the bait plasmid

To determine whether the bait plasmid had the ability to be autoactivated or be toxic, the bait plasmid and the pGBKT7 vector (empty vector) were individually transformed into Y2H Gold competent cells based on the user manual of Quick & Easy Yeast Transformation Mix (Clontech, California, USA). Subsequently, the transformants were cultured on SD/-Trp (SDO), SD/-Trp/X-α-Gal (SDO/X) and SD/-Trp/X-α-Gal/AbA (SDO/X/A) agar plates at 30 °C for 3–5 days. Only the colonies grown on SDO and SDO/X plates were white or very pale blue, and no colonies on SDO/X/A plates were observed. The bait plasmid was determined to not be autoactivated. If the bait plasmid was toxic, the size of the colonies would have been remarkably smaller than that of the colonies containing the empty vector plasmid. The bait plasmid, which exhibited neither toxicity nor autoactivation, could be used in the following Y2H screening experiment.

### Y2H screening

A Y2H screening system was carried out to screen the interacting proteins between recombinant SVSP454-pGBKT7 (bait plasmid) and the cDNA library of bovine B cells (prey plasmid) [16]. The bait plasmid and prey plasmid were cotransformed into Y2H Gold competent cells at the library scale using Yeastmaker™ Yeast Transformation System 2 (Clontech, California, USA) according to the user manual. The transformants were then plated on DDO/X/A agar plates and incubated at 30 °C for 5 days. The blue colonies were placed again on the DDO/X/A agar plates for one more screening to eliminate false-positive colonies. Finally, the blue colonies from the second screening were picked and incubated on QDO/X/A agar plates at 30 °C for 3–5 days. The blue colonies were cultured onto QDO/X/A agar plates again. Moreover, pGADT7-T and pGBKT7-Lam, which were used as negative controls, were cotransformed into Y2H Gold cells. The pGADT7-T and pGBKT7-53 plasmids served as positive controls and were cotransformed into Y2H Gold cells. Both cotransformants were plated onto DDO and DDO/X/A agar plates at 30 °C for 3–5 days.

After Y2H system screening, colony PCR was performed with Matchmaker™ Insert Check PCR Mix (Clontech, California, USA) for identification of the blue colonies.

### Rescue of the putative prey plasmids

The putative prey plasmids were extracted from the identified blue colonies using an Easy Yeast Plasmid Isolation Kit (Clonetech, California, USA) based on the user manual. Subsequently, 3 μL of purified prey plasmids was transformed into *E. coli* DH5α competent cells for plasmid DNA extraction and sequencing analysis.

### Positive prey gene analysis

Corresponding bovine genes were identified after analyzing the sequences of putative prey plasmids with the BLAST function in NCBI [17]. The protein construction and function of the confirmed bovine genes were analyzed through the UniProt database (http://www.uniprot.org/) and SMART (http://smart.embl-heidelberg.de/).

### Expression and subcellular localization of the bait and prey proteins

The recombinant p3 × FLAG-CMV-prey plasmids were constructed by cloning prey genes into the p3 × FLAG-CMV vector plasmid, which had been digested with the restriction enzymes *HindIII*/*BamHI* (Thermo Fisher Scientific, #FD0504 and #FD0054) or *HindIII*/*XbaI* (Thermo Fisher Scientific, #FD0504 and #FD0684). The pcDNA3.1 + -bait-MYC recombinant plasmid was constructed by cloning the bait gene with a C-terminal MYC tag into the *BamHI*/*XhoI* (Thermo Fisher Scientific, #FD0054 and #FD0694) digested pcDNA3.1 + vector plasmid. HEK293T cells were seeded on glass slides in 6-well cell culture plates at a density of 5 × 10^5^ cells/ml. When cells became 70–90% confluent, the bait and/or prey plasmids were transfected or cotransfected into HEK293T cells using Lipofectamine 3000 (Thermo Fisher Scientific, #L3000015). Moreover, empty vectors (p3 × FLAG-CMV and pcDNA3.1+) serving as negative controls were also transfected into the cells. After 24 h of transfection, the cells were fixed with 4% paraformaldehyde for 30 min at RT. Following washing with PBS, the cells were permeabilized with 0.1% Triton X-100 for 15 min at RT. The cells were blocked with 3% BSA in PBS at RT for 1 h after washing. For the immunofluorescence assay, the cells were incubated with a 1:200 diluted anti-MYC tag monoclonal antibody derived from mouse (CST, #2276S) or a rabbit anti-FLAG tag monoclonal antibody (Sigma, #F7425) at 4 °C overnight. After five washes with PBS, the cells were stained with goat anti-mouse Alexa Fluor 488-or donkey anti-rabbit 594-conjugated secondary antibodies (Life Technologies, USA) at a 1:1000 dilution in PBS with 3% BSA for 1 h at RT. Following the washing step, the cells were incubated with 1:2000 diluted Hoechst 33342 (Life Technologies, USA) for nuclear staining at RT for 15 min. The cells were then observed under a confocal microscope (Leica TCS) with a 63 × oil objective. For each slide, more than 100 random cells were examined, and the presented images for each slide we selected were the most representative in the present study.

### Coimmunoprecipitation (Co-IP) assay

HEK293T cells were seeded in 10-cm cell culture dishes (Thermo Fisher Scientific) at 2 × 10^6^ cells per dish. After culturing for 14 h, 10 µg of each constructed bait plasmid and the constructed prey plasmids were cotransfected into the cells. After 48 h, the cells were washed with cold PBS twice and lysed with 600 μL of IP/lysis buffer (Thermo Fisher Scientific) on ice containing phosphatase inhibitor cocktail (Roche, #4906845001) and protease inhibitor (Roche, #4693132001). Then the cell lysates were centrifuged at 16,000×*g* at 4 °C for 10 min. The immunoprecipitation experiment was performed with mouse anti-FLAG tag monoclonal antibody (Sigma, #F1804) using a Pierce™ Co-Immunoprecipitation kit (Thermo Fisher Scientific, #26149), and the procedure was performed based on the protocol provided by the kit. Next, empty vectors (p3 × FLAG-CMV and pcDNA3.1+) which served as negative controls, were also cotransfected into HEK293T cells. The elution of the Co-IP was subjected to western blotting and checked with the mouse anti-FLAG tag monoclonal antibody and rabbit anti-MYC tag monoclonal antibody (CST, #2278S).

### Bimolecular fluorescence complementation (BiFC) assay

BiFC analysis is a technically straightforward assay for the visualization of protein interactions in living cells by fluorescence microscopy or flow cytometry. The BiFC method is based on the association between two nonfluorescent complementary fragments of Venus, a variant of green fluorescent protein (GFP). Two Venus fragments are fused to the bait and prey proteins (an interacting protein pair), and when they are coexpressed in the same cells and interact, they are brought in close proximity to each other, leading to structural complementation as well as a bright fluorescent signal [18]. Moreover, the assay can be used to observe the subcellular localization of the interacting proteins [18].

pBiFC-VN173 (Addgene, 22010) and pBiFC-VC155 (Addgene, 22,011) vectors were obtained from Addgene [19]. The pBiFC-VN173-bait plasmid and prey plasmids were constructed by cloning bait or prey genes into the *Hind*-*III*/ *Sal*-*I* (Thermo Fisher Scientific, #FD0504 and #FD0644) digested pBiFC-VN173 vector. pBiFC-VC155-bait/prey plasmids were generated by cloning the bait or prey genes into the pBiFC-VC155 vector after *Sal*-*I*/*Kpn*-*I* (Thermo Fisher Scientific, #FD0644 and #FD0524) digestion. The pBiFC-VN173-bait and its prey plasmids, and pBiFC-VC155-bait and its prey plasmids were transfected individually into HEK293T cells. Moreover, the pairs of pBiFC-VN173-bait/pBiFC-VC155-prey or pBiFC-VN155-bait/pBiFC-VC173-prey were cotransfected into HEK293T cells using Lipofectamine 3000. In addition, the empty pBiFC-VN173 and pBiFC-VC155 vectors served as the controls and were also transfected into HEK293T cells. After 24 h of transfection, confocal experiments were performed as described above. After washing, fixing, permeabilizing, and blocking, the cells were stained with 100-fold diluted rabbit anti-HA tag monoclonal antibody (CST, #3724S) or 200-fold diluted mouse anti-FLAG tag monoclonal antibody by incubation at 4 °C overnight. After washing, the cells were incubated with goat anti-mouse Alexa Fluor 594- or donkey anti-rabbit 594-conjugated antibodies (Life Technologies, USA) at a dilution of 1:1000 at RT for 1 h. After staining the cell nuclei with Hoechst 33342 (Life Technologies, USA), the cells were examined under confocal microscope (Leica TCS).

### Flow cytometry

HEK293T cells (5 × 10^6^ cells/ml) were cultured in 6-well culture plates (Corning, USA). The pBiFC-VN173-bait/prey plasmids and the pBiFC-VC155-bait/prey plasmids were transfected individually into HEK293T cells when the cells were 70–90% confluent. Moreover, the pairs of pBiFC-VN173-bait/pBiFC-VC155-prey and pBiFC-VN155-bait/pBiFC-VC173-prey were cotransfected into HEK293T cells when the density of cells was 70–90% confluent. After 48 h of transfection, the Venus fluorescence of transfected cells was checked with fluorescence microscopy. Following two washes with PBS, the cells were digested with trypsin–EDTA (0.25%) (Gibco, New York, USA). The cells were then washed with cold PBS and centrifuged at 800 rpm for 3 min at 4 °C to remove the remaining trypsin–EDTA. Finally, the cell samples were resuspended in cold PBS, and the mean fluorescence intensity (MFI) was detected by using a BD Accuri™ C6 Plus Flow Cytometer (USA).

### Data and statistical analyses

GraphPad Prism 7 was used for statistical analysis in our study. The results represent the mean ± standard error of three repeated independent experiments for all figures. Statistical analysis was performed using unpaired two-tailed Student’s *t*-tests to determine the significance of differences between groups. NS, not significant (*p* > 0.05), *p < 0.05, **p < 0.01, and ***p < 0.001.

## Results

### Transcriptional profiles of SVSP950454 at the mRNA level

**Fig. 1:**
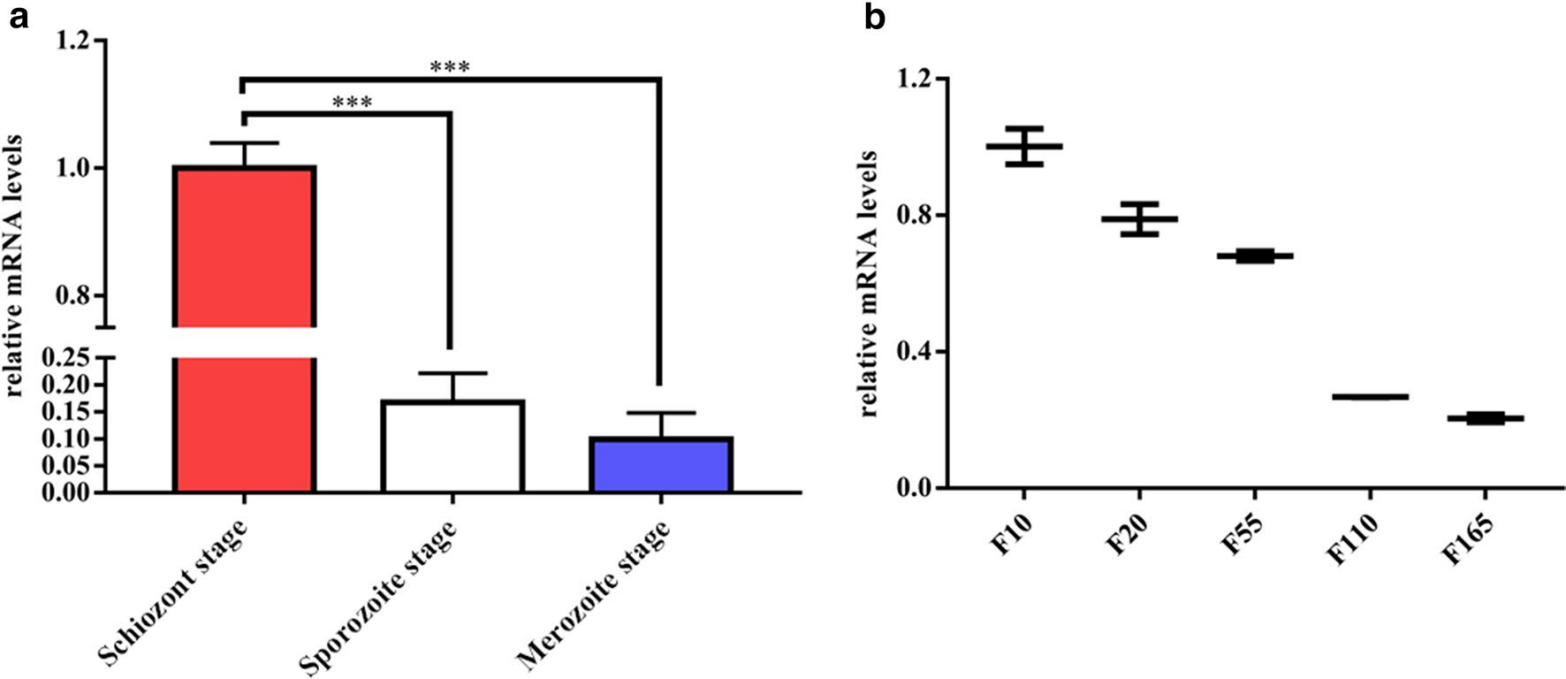
Transcriptional profiles of SVSP950454 at the mRNA level. **a** mRNA levels of SVSP454 at different stages of *T. annulata*. **b** mRNA levels of SVSP454 in different cell culture passages of *T. annulata-*infected cells

As shown in Fig. 1a, the SVSP454 gene was mainly transcribed at the *T. annulata* schizont stage, in accordance with the findings of previous studies [11, 15].

The qPCR results indicated that the transcription level of SVSP454 was gradually reduced from passages F10 to F165 of *T. annulata-*infected cells (Fig. 1b).

### Colocalization of the SVSP454 in *T. annulata-*infected cells

**Fig. 2:**
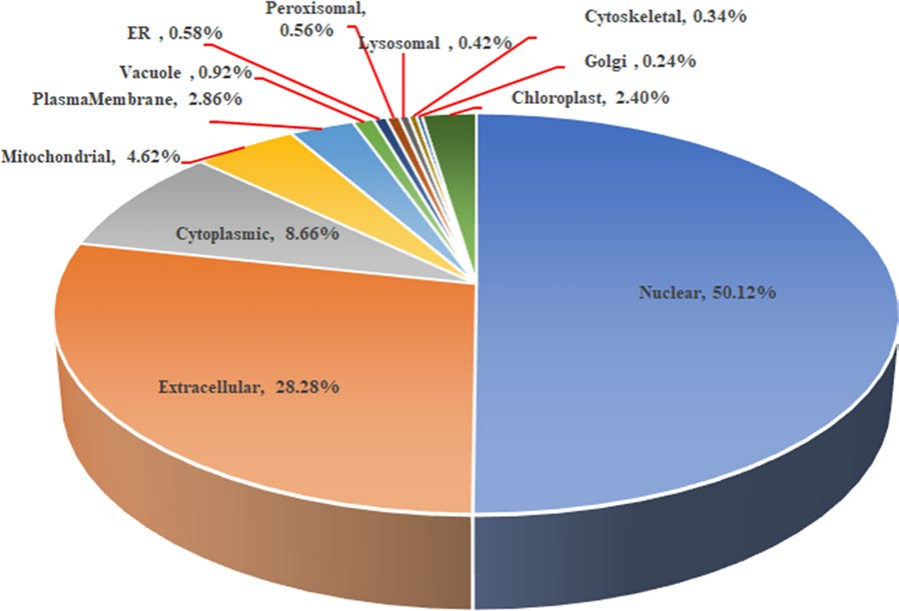
Schematic of the subcellular location of SVSP950454 using CELLO v.2.5. ER represents the endoplasmic reticulum

**Fig. 3:**
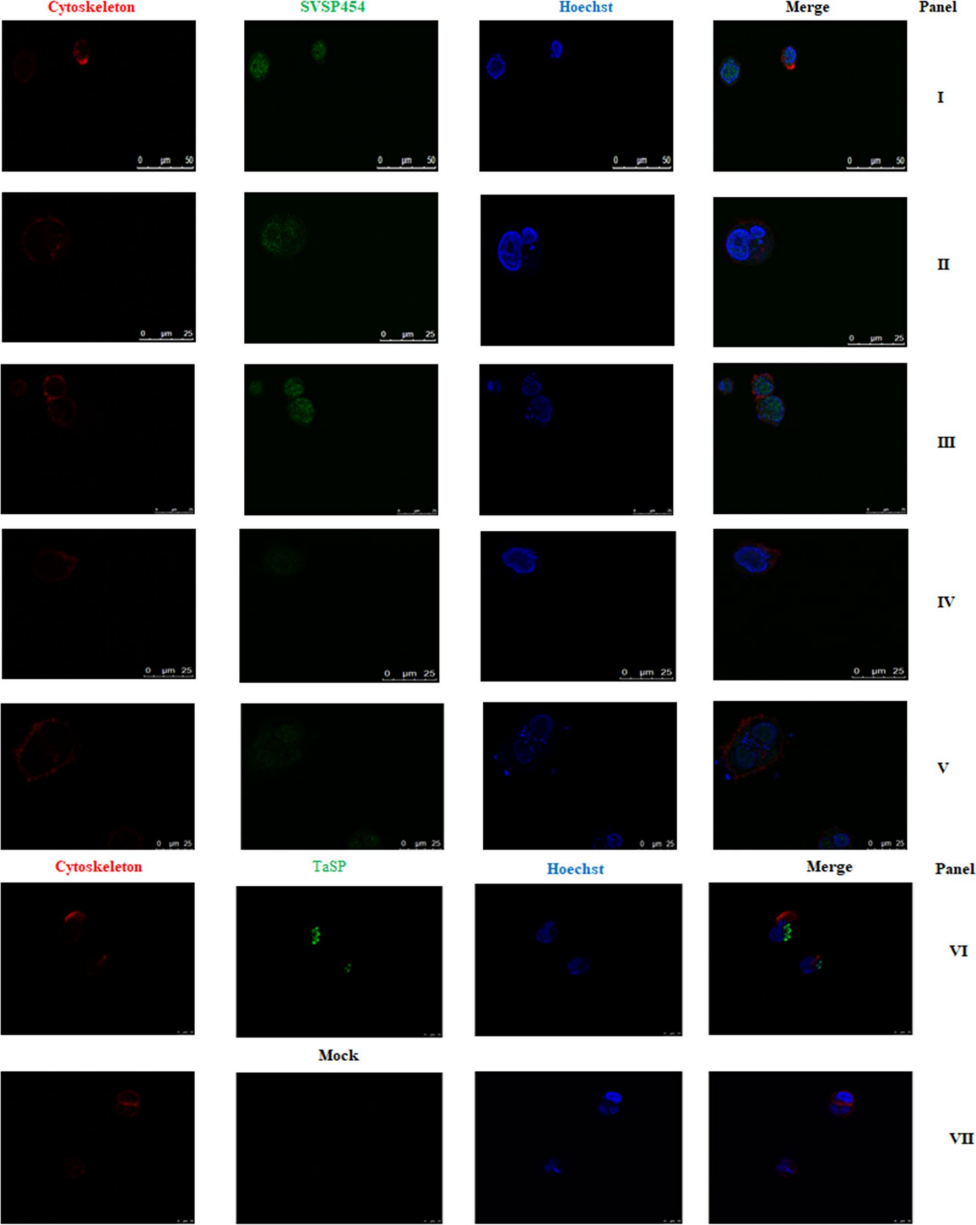
Subcellular distribution of the SVSP454 protein in *T. annulata*-infected cells. Panels I to V represent the subcellular distribution of the SVSP454 protein in the corresponding cell culture passages (F10, F20, F55, F110, and F165) of *T. annulata-*infected cells. Panels VI and VII show the positive and negative controls in *T. annulata-*infected cells, respectively. SVSP454 proteins (panel I to V) and TaSP proteins (panel VI) were stained with rabbit anti-SVSP454 and TaSP polyclonal antibodies, which were each diluted at a ratio 1:100, followed by incubation of the cells with goat anti-rabbit antibody conjugated with Alexa Fluor 488 at a1:1000 dilution. Hoechst 33342 was used at a 1:2000 dilution to label the cell nucleus, and the cytoskeleton was stained with Alexa Fluor™ 594 phalloidin at a dilution of 1:100. Subcellular localization of SVSP454 was observed by confocal microscopy

With the analysis results from CELLO v.2.5, SVSP454 was determined to be mainly distributed in the nucleus and extracellular space of *T. annulata*-infected cells (Fig. 2). To further confirm the specific cellular distribution of the SVSP454 protein in *T. annulata-*infected cells, a confocal microscopy assay was carried out (Fig. 3). The findings of the confocal experiment were consistent with the results of CELLO v.2.5. Moreover, the protein was mainly distributed in the cytoplasm and nucleus of *T. annulata*-infected cells at different passages, including F10, F20, F50, F110, and F165.

### Construction and identification of the bait plasmid

**Fig. 4:**
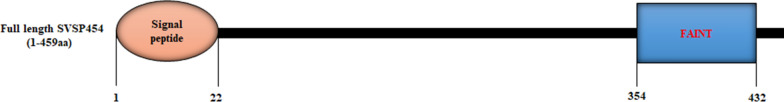
Gene structure of SVSP454 (bait plasmid) by SMART analysis

The SVSP454 gene was obtained from the cDNA of *T. annulata*-infected cells. The ORF length of the SVSP454 gene was 1377 bp, which encoded 459 amino acids, and the molecular weight of the protein was approximately 54 kDa. However, the ORF of the reference gene was 1287 bp, encoding 429 aa. The sequence identity of nucleotides and amino acids of the obtained sequence with the reference sequence was 88.9% and 80.2%, respectively. In addition, the protein was determined by bioinformatics analysis to contain a signal peptide (1–22 aa), and a FAINT domain (354–432 aa), which are the typical regions found in the SVSP family of *T. annulata* (Fig. 4)

### Determination of autoactivation and toxicity of bait plasmid

**Fig. 5:**
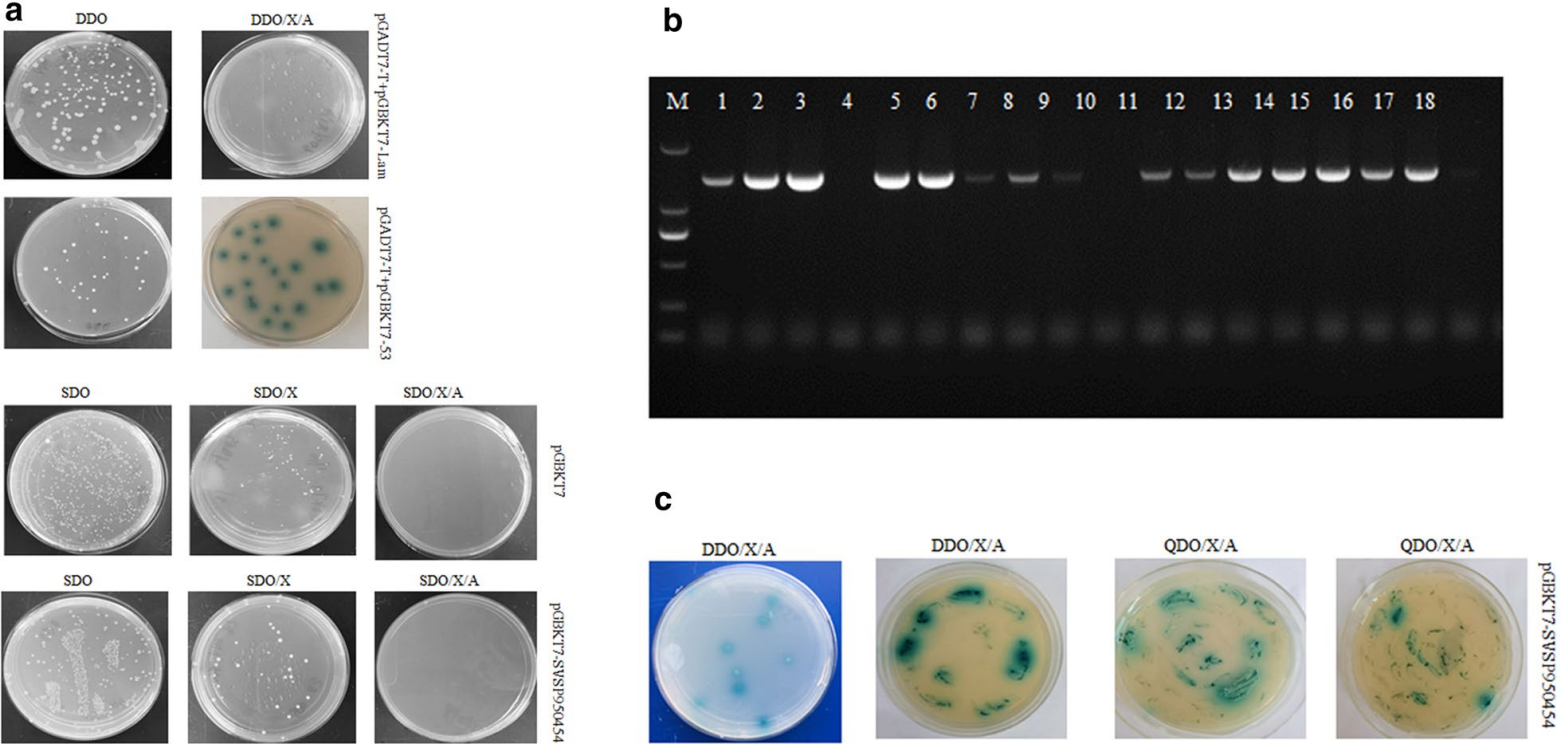
Screening of interacting proteins with SVSP454 using the Y2H system. **a** Detection of the autoactivation ability and toxicity of the bait plasmid (pGBKT7-SVSP454) in Y2H Gold cells. **b** Identification of the transformation efficiency of the pGBKT7-SVSP454 bait plasmid. The colonies were randomly selected and identified by colony PCR using the specific primers for SVSP454. M: DL 2000 DNA marker; 1–18: colonies grown on SDO plates with SVSP454 bait plasmid. **c** Screening for the putative prey proteins interacting with the SVSP454 bait plasmid. The cotransformants of bait and prey plasmids were successively grown on DDO/X/A and QDO/X/A plates. The SVSP950454 bait and pGBKT7 empty plasmids were transformed into Y2H Gold cells and cultured on different plates. Negative control (pGADT7-T and pGBKT7-Lam) plasmids and positive control (pGADT7-T and pGBKT7-53) plasmids were cotransformed into Y2H Gold cells and incubated on DDO and DDO/X/A plates at 30 °C

Before Y2H screening, we determined the autoactivation ability and toxicity of the bait plasmid. The results showed that the sizes of the colonies for the SVSP454 bait plasmid grown on the SDO and SDO/X plates were similar to those of the colonies with the empty vector incubated on the same plates (Fig. 5a). Therefore, the findings indicate that the bait plasmid was not toxic. Moreover, the color of the colonies containing the bait plasmid was white and prey blue when cultured on SDO and SDO/X plates (Fig. 5a), which shows that the bait plasmid was not autoactivated in the Y2H screening system. The transformation efficiency of SVSP454 was 88.89% based on the specific PCR results (Fig. 5b).

### Y2H screening

We used the Y2H system to screen the potential proteins that interact with SVSP454. The blue colonies of the bait and prey plasmid transformants cultured on DDO/X/A plates were continually picked and incubated on DDO/X/A and QDO/X/A plates for further screening of the possible proteins (Fig. 5c). The obtained potential interacting prey plasmids were identified by sequencing analysis and prey plasmid rescue.

### Analysis of the putative prey proteins

To identify the nucleotide sequence of the potential prey proteins interacting with SVSP454, two obtained prey plasmids were sequenced using the specific primers pGADT7-F/R. The nucleotide fragments were analyzed using the BLAST Tool in NCBI. The fragment of one prey plasmid has 99.69% identity with *Bos Taurus* coiled-coil domain 181 (CCDC181, NM_001205801.1). The sequence corresponded to CCDC181 from 118 to 1089, which encoded 327 amino acids (aa). UniProt and STRING analysis [20] showed that CCDC181 is a microtubule-binding protein that localizes to the microtubular manchette of elongating spermatids and contains two coiled-coil domains at residues 45–65 and 415–435, which are related to the microtubule-binding function. For another prey plasmid, the similarity of its nucleotide sequence with *Bos Taurus* mitochondrial ribosomal protein L30 (MRPL30, BC_112734.1) was 99.08%. The sequence of the latter prey plasmid encoded 161 aa of the complete MRPL30 CDS regions. MRPL30 belongs to the universal ribosomal protein UL30 family, containing a transit peptide (1–34 aa) domain. In terms of its biological process, MRPL30 is involved in translation and structural constituent ribosomes, as shown in the UniProt and STRING analyses.

### Expression and subcellular localization of SVSP454 and its potential interacting proteins

**Fig. 6:**
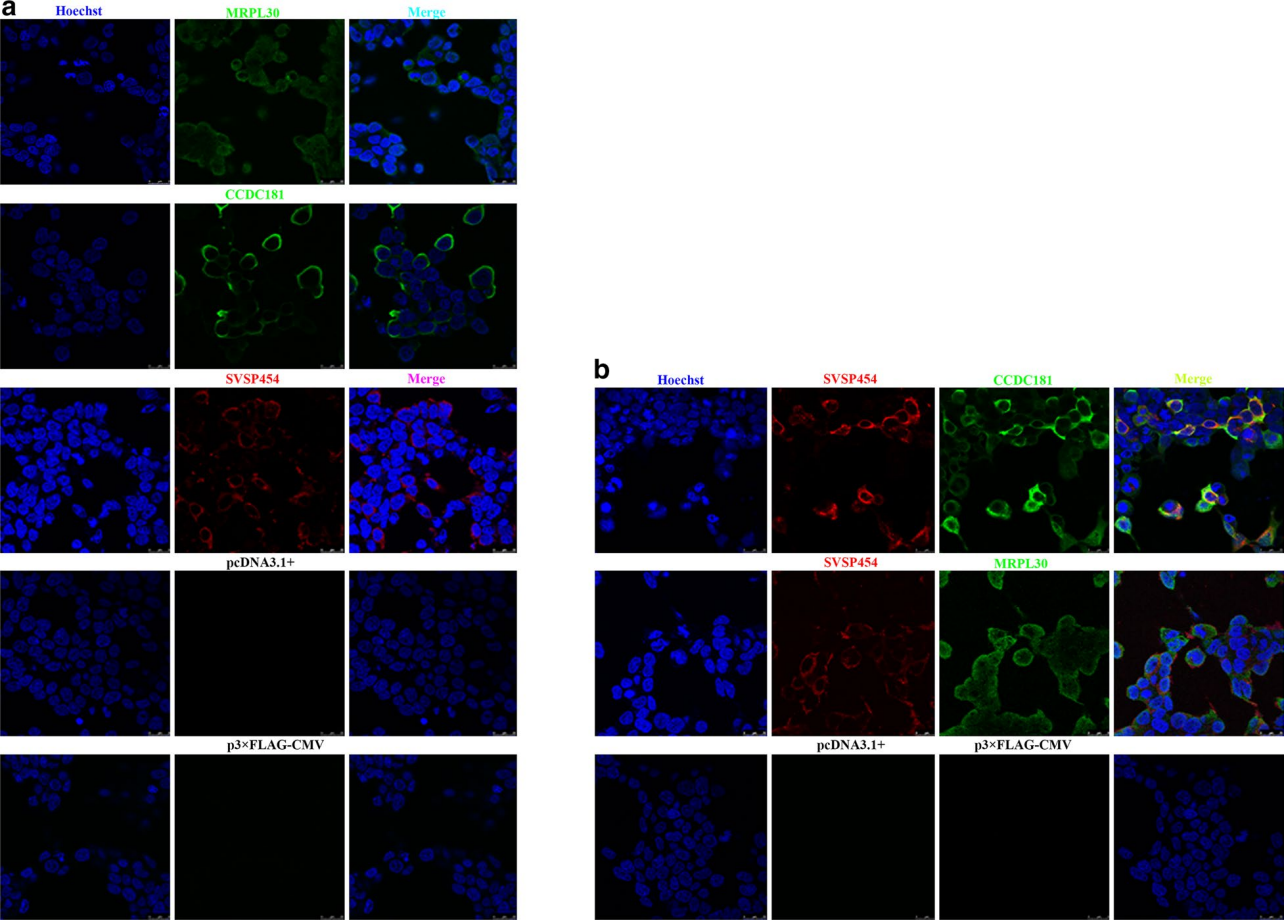
Expression and subcellular localization of SVSP454 and its prey proteins (CCDC181 and MRPL30) in HEK293T cells. SVSP454 (4 µg) and its potential interacting proteins-CCDC181 (4 µg) and MRPL30 (4 µg) were individually **a** or pairwise (SVSP454 (2 µg)-CCDC181 (2 µg) and SVSP454 (2 µg)-MRPL30 (2 µg)), **b** transfected into HEK293T cells, stained with a mouse anti-MYC tag monoclonal antibody or rabbit anti-FLAG tag monoclonal antibody, and incubated with a goat anti-mouse Alexa Fluor 488- or donkey anti-rabbit 594-conjugated antibody. Cell nucleus was stained with Hoechst 33342

Before the Co-IP assay was performed, recombinant plasmids of MYC-tagged SVSP454 and its potential binding proteins (FLAG-tagged CCDC181 and FLAG-tagged MRPL30) were successfully obtained. Then, MYC-tagged SVSP454, FLAG-tagged CCDC181, and FLAG-tagged MRPL30 were expressed individually or pairwise in HEK293T cells (Fig. 6a, b). Subcellular localization of these proteins was observed in the cell cytoplasm.

### Detection of the interaction of SVSP454 with its prey protein by Co-IP

**Fig. 7:**
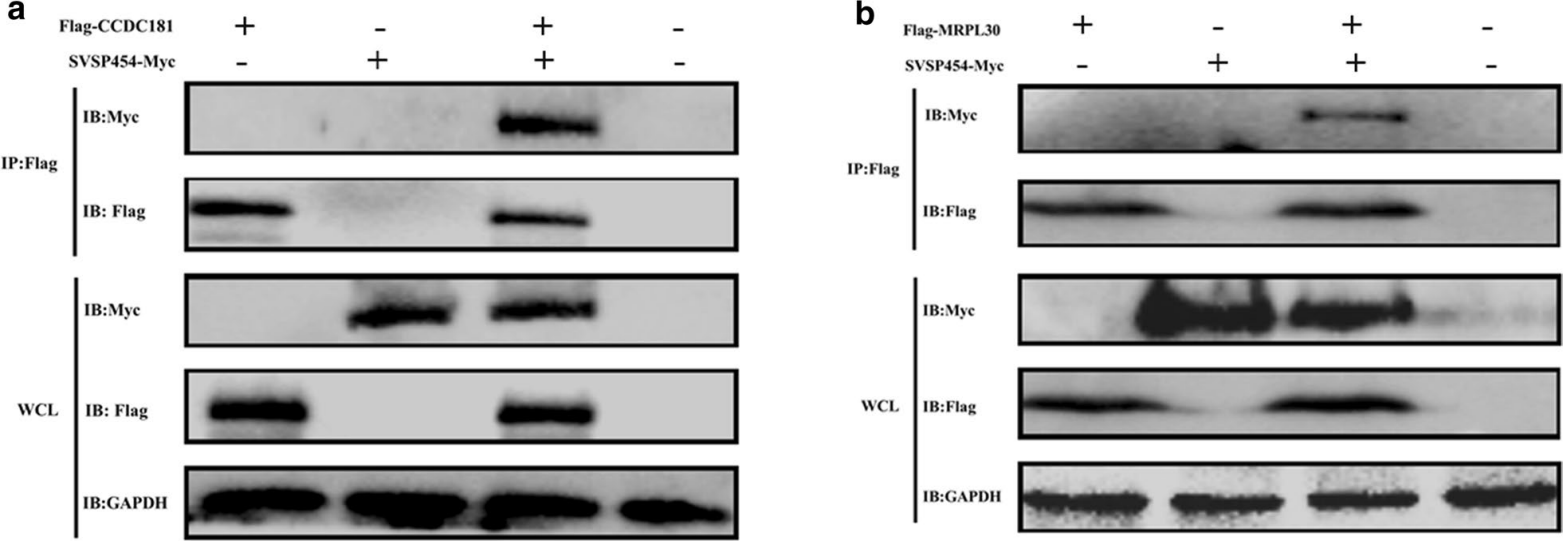
SVSP454 interacts with both CCDC181 and MRPL30. Coimmunoprecipitation (Co-IP) and immunoblotting (IB) identification of HEK293T cells expressing SVSP454-MYC (10 µg) and its potential interacting protein FLAG-tagged CCDC181 (10 µg) (**a**) and SVSP454-MYC (10 µg) along with FLAG-tagged MRPL30 (10 µg) (**b**). WCL represents IB analysis of whole HEK293T cell lysates without IP

To confirm the aforementioned interactions, we transfected MYC-tagged SVSP454 and its potential interacting proteins (FLAG-tagged CCDC181 and FLAG-tagged MRPL30) into HEK293T cells and demonstrated that both CCDC181 and MRPL30 interacted with SVSP454, as shown by the results of the Co-IP assays (Fig. 7a, b).

### SVSP454 interacts directly with CCDC181 and MRPL30 in cells

**Fig. 8:**
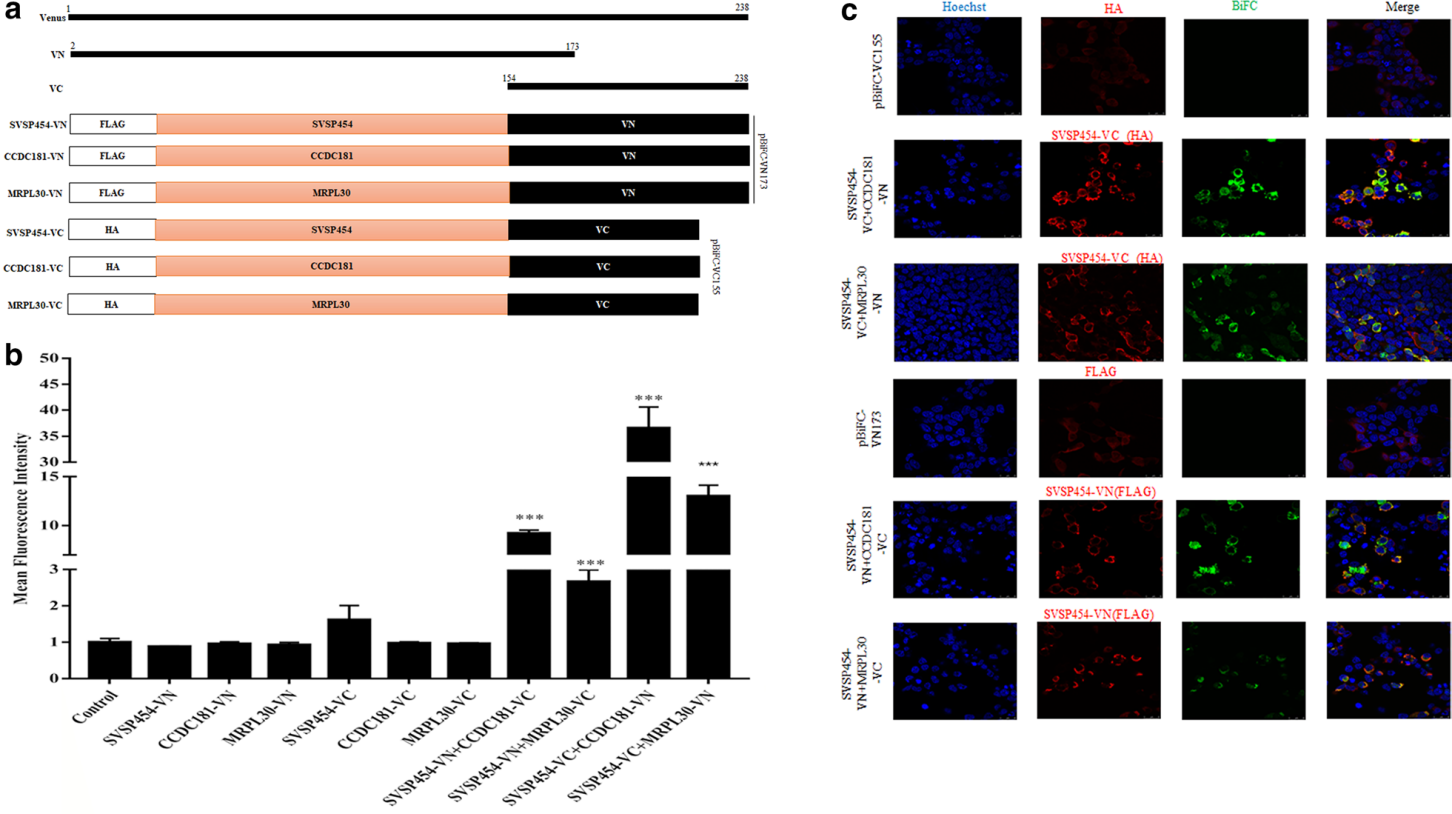
SVSP454 binds to both CCDC181 and MRPL30 in the cell context. **a** Schematic of recombinant plasmid construction for the BiFC assays. VN and VC were fused to the C-terminus of SVSP454 and its interacting proteins (CCDC181 and MRPL30), and each fusion protein also contained a FLAG or HA tag at its N-terminus. **b** SVSP454-VN, SVSP454-VC, CCDC181-VN, CCDC181-VC, MRPL30-VN, MRPL30-VC, SVSP454-VN-CCDC181-VC, SVSP454-VN-MRPL30-VC SVSP454-VC-CCDC181-VN and SVSP454-VC-MRPL30-VN were expressed individually or pairwise in HEK293T cells as indicated. Four micrograms of plasmids were transfected into HEK293T cells. The MFI of BiFC fluorescent signals was detected by flow cytometry and is presented as MFI values relative to the signal of the control HEK293T cells. Error bars represent the SD from three independent BiFC experiments. **c** HEK293T cells were cotransfected with the pairs SVSP454-VN-CCDC181-VC (4 μg), SVSP454-VN-MRPL30-VC (4 μg), SVSP454-VC-CCDC181-VN (4 μg) and SVSP454-VC-MRPL30-VN (4 μg). Empty vectors pBiFC-VN173 (4 μg) and pBiFC-VC155 (4 μg) served as the negative control and were individually transfected into HEK293T cells

To further examine the interactions of SVSP454 with CCDC181 and MRPL30 in a cellular context, BiFC assays were performed to investigate how SVSP454 is targeted by its interacting proteins. N-terminal residues 2 to 173 of Venus (VN), which is in pBiFC-VN173, and C-terminal residues 154 to 238 (VC), which is in pBiFC-VC155, were fused to the C-terminus of SVSP454 or CCDC181 and MRPL30, respectively (Fig. 8a). When SVSP454-VN, SVSP454-VC, CCDC181-VN, CCDC181-VC, MRPL30-VN, and MRPL30-VC were expressed individually in HEK293T cells, no fluorescent signals were observed by flow cytometry (Fig. 8b). However, when SVSP454-VN or SVSP454-VC were paired with complementary CCDC181-VC or CCDC181-VN and MRPL30-VC or MRPL30-VN, respectively, strong fluorescent signals were detected (Fig. 8c).

To investigate the specificity and subcellular colocalization of SVSP454-CCDC181 and SVSP454-MRPL30, the BiFC fluorescent signals were visualized through confocal microscopy. When the pairs SVSP454-VN/CCDC181-VC and SVSP454-VC/MRPL30-VN were expressed, strong green BiFC fluorescent signals were detected, which was consistent with the flow cytometry results (Fig. 8b, c). The green BiFC signals colocalized with the red fluorescent signals from the anti-FLAG or HA-tagged antibodies of SVSP454 (Fig. 8c) showed perinuclear subcellular localization in HEK293T cells. These results demonstrate that SVSP454 interacted with both CCDC181 and MRPL30 in the intracellular compartments.

### Discussion

*Theileria annulata* causes acute infection in bovines, leading to a huge economic burden in the regions where it occurs. It can transform host cells and cause cancer-like phenotypes. A captivating question is which *T. annulata* molecules contribute to these host cell cancer hallmarks. Therefore, investigating the interactions of *T. annulata* and its host provides insights into *T. annulata*-induced pathogenesis. Although some molecules have been reported to affect *T. annulata*-induced transformation, metabolism, and invasion by regulating host signaling-hubs [8–10, 21], the evidence for the relevant roles of secreted multifamily (SVSP)-host interactions has not been presented until now. Herein, SVSP454, a member of the SVSP multigene family, was used as bait to explore its interacting molecules in host cells.

Although SVSP454 is expressed at the schizont, sporozoite, and merozoite stages of *T. annulata*, the expression level at the schizont stage of *T. annulata* is the highest (Fig. 1a), which is consistent with previous results [11, 15]. Of particular interest, the transcription level of SVSP454 was significantly reduced with increasing passages of *T. annulata*-transformed cells (Fig. 1b). Therefore, we propose that it is likely involved in the virulence and transformation of *T. annulata* by interfering with some host cell proteins. Furthermore, the SVSP family has been suspected to be related to immune evasion [14]. The subcellular distribution of SVSP454 was mainly in the cell nucleus and extracellular regions of *T. annulata* infected cells. These findings suggest that SVSP454 may perform its specific function by interacting with host proteins in *T. annulata* schizont-transformed cells.

The most likely explanation for only 80–88% sequence identity with the reference SVSP454 gene is that the reference genome was obtained from the C9 clone of the Ankara first isolated in Turkey [11], whereas the SVSP454 sequence we obtained was determined for a parasite-infected line first isolated at Xinjiang, China. Therefore, it is important to identify the host proteins that interact with SVSP454 in the present study. The results show that the potential prey plasmids interacting with SVSP454 are CCDC181 and MRPL30 from bovines after sequencing and BLAST analyses. To further determine whether SVSP454 interacts with the screened proteins by the Y2H system, the coimmunoprecipitation (Co-IP) assay was performed to identify the interactions between SVSP454 and its prey proteins. Prior to performing Co-IP, we constructed recombinant plasmids containing MYC or FLAG tagged for bait and prey plasmids, and they were individually or pairwise expressed in HEK293T cells. Fluorescence was observed by confocal microscopy (Fig. 6). The Co-IP results demonstrate that SVSP454 interacted with both CCDC181 and MRPL30 (Fig. 7). Furthermore, the interactions of the SVSP454-MRPL30 and SVSP454-CCDC181 pairs were confirmed by a BiFC assay in living cells, and the subcellular colocalization of the interacting pairs was investigated in intracellular compartments of HEK293T cells (Fig. 8b, c). Therefore, in the present study, SVSP454 was found to interact with both CCDC181 and MRPL30 by both Co-IP and BiFC assays.

CCDC181, coiled-coil domain 181 of bovine, is a microtubule binding protein that contains two coiled-coil domains at residues 45–65 and 415–435. Although its molecular function is not clear, some studies have indicated that it interacts with HOOK1 in haploid male germ cells [22], and it is a methylation-driven gene associated with the progression and prognosis of lung adenocarcinoma [23, 24], human papillomavirus-related oropharyngeal squamous cell carcinoma [25], prostate cancer [26–29], and breast cancer [30]. Because *T. annulata*-transformed host cells have cancer hallmarks, we propose that CCDC181 might be used as a biomarker for tropical theileriosis in future studies. Moreover, as a microtubule-binding protein, the interactions between SVSP454 and CCDC181 are potentially involved in microtubule network dynamics during host cell mitosis, which is similar to findings regarding TaSP [31] and TaMISHIP [32], which were formed an interacting network by interacting with CLASP1, EB1, CD2AP to regulating the host cell cytoskeleton. Therefore, the present results show that SVSP454 has the potential function of regulating host microtubule dynamics by stabilizing CCDC181 and possesses an SVSP454-CCDC181-microtubule axis to hijack host cells for proliferation.

Another identified protein, mitochondrial ribosomal protein L (MRPL30), belongs to the universal ribosomal protein UL30 family. Its function may be involved in translation and ribosome structure according to UniProt and STRING analyses. Until now, there have been few studies on MRPL30, but one study reported that it could be a candidate gene for type 2 diabetes mellitus and obesity [33]. In the present study, we demonstrated that SVSP454 is directly bound to MRPL30. Although the biological process involving MRPL30 is not clear, our findings will pave the way to study how the SVSP454-MRPL30 interactions regulate the host signaling and parasite survival in future research.

## Conclusion

SVSP454, a member of the SVSP multigene family, was used as a bait plasmid, and its prey proteins CCDC181 and MRPL30 were screened out by using the Y2H system. Then, we demonstrated that SVSP454 directly interacted with both CCDC181 and MRPL30 by the Co-IP and BiFC assays. Therefore, we speculate that SVSP454-CCDC181/SVSP454MRPL30 is an interacting axis that regulates the microtubule network and translation process of the host by some vital signaling molecules. However, Y2H system screening was only performed once, and some other interacting host proteins might be missed during our study. In future studies, we will continue to explore new SVSP454 interacting proteins. Unfortunately, reports about the functions of CCDC181 and MRPL30 are limited, which provides few opportunities to investigate the molecular mechanism of SVSP454-CCDC181 and SVSP454-MRPL30 interactions.

## Data Availability

The datasets supporting the conclusions of this article are included within the article.

## Abbreviations

SD/-Trp (SDO): Synthetically defined medium without tryptophan
SD/-Trp/X-α-Gal (SDO/X): Synthetically defined medium without tryptophan supplemented with X-α-Gal
SD/-Trp/X-α-Gal/AbA: Synthetically defined medium without tryptophan supplemented with X-α-Gal
(SDO/X/A): Synthetically defined medium without tryptophan and leucine supplemented with X-α-Gal and aureobasidin A
DDO: Synthetically defined medium without tryptophan and leucine
DDO/X/A: Synthetically defined medium without tryptophan and leucine supplemented with X-α-Gal and aureobasidin A
QDO/X/A: Synthetically defined medium without tryptophan, leucine, adenine, histidine supplemented with X-α-Gal and aureobasidin A
